# Centenarians as Models of Resistance and Resilience to Alzheimer’s Disease and Related Dementias

**DOI:** 10.20900/agmr20200018

**Published:** 2020-07-03

**Authors:** Stacy L. Andersen

**Affiliations:** Department of Medicine, Boston University School of Medicine, Boston, MA 02118, USA

**Keywords:** centenarian, longevity, health span, dementia, resilience, resistance, neurodegenerative

## Abstract

The majority of research to understand the pathogenesis of and contributors to Alzheimer’s disease (AD) pathology, dementia, and disease progression has focused on studying individuals who have the disease or are at increased risk of having the disease. Yet there may be much to learn from individuals who have a paradoxical decreased risk of AD suggesting underlying protective factors. Centenarians demonstrate exceptional longevity that for a subset of the cohort is associated with an increased health span characterized by the delay or escape of age-related diseases including dementia. Here, I give evidence of the association of exceptional longevity with resistance and resilience to AD and describe how cohorts of centenarians and their offspring may serve as models of neuroprotection from AD. Discoveries of novel genetic, environmental, and behavioral factors that are associated with a decreased risk of AD may inform the development of interventions to slow or prevent AD in the general population. Centenarian cohorts may also be instrumental in serving as controls to individuals with AD to identify additional risk factors.

## INTRODUCTION

Most clinical research on Alzheimer’s disease (AD) has focused on studying individuals at various stages of the disease from preclinical AD to severe dementia as defined by clinical signs and symptoms, cognitive dysfunction, or biomarker evidence of amyloid, tau, and neurodegeneration. An alternative to studying individuals with AD is to study individuals who demonstrate either resistance to AD (i.e., those who avoid both the cognitive and pathological markers of AD) or resilience to AD (i.e., those who have significant AD pathology yet maintain good cognitive function) [1]. This will allow us to better understand how protective factors such as genetics and behavioral and lifestyle differences contribute to the risk and pathogenesis of Alzheimer’s disease and related dementias.

Centenarians, individuals who have reached the age of 100 years, demonstrate exceptional longevity because they have surpassed the top 1 percentile of survival of their birth cohort. Yet, perhaps more importantly, many centenarians also have exceptional health spans characterized by the delay or escape of age-related diseases [2]. This is surprising since age is a major risk factor for many chronic diseases including neurodegenerative diseases (see [3] for a review). Therefore, centenarians, who have an increased risk of AD due to their advanced age, but are able to delay or avoid dementia and AD pathologic changes may be an informative cohort for learning about mechanisms of resistance and resilience to AD and related dementias.

## EPIDEMIOLOGY OF DISEASE AND DEMENTIA AMONG CENTENARIANS

Although human life expectancy has increased over the past two decades, individuals in most countries do not appear to be living healthier. Disease prevalence, disability, and the number of years spent with disease or disability have all increased [4,5]. Yet in contrast, many centenarians, do not follow this trend. Rather, exceptional longevity is associated with a reduced risk of morbidity and, on average, a delay in the onset of age-associated diseases including cancer, cardiovascular disease, stroke, and dementia [2,6]. Throughout older adulthood, in comparison to their peers who do not survive to 100 years, centenarians have fewer diseases and limitations in performing activities of daily living [7] and are less likely to be hospitalized [8]. Moreover, living to extreme ages has been associated with compression of morbidity and disability [9], or shortening the proportion of life spent with disease and disability toward the end of life. In fact, supercentenarians, individuals aged 110+ years, spend only 5% of their lives on average with an age-related disease in comparison to 18% for younger controls [2] with many maintaining functional independence up to the age of 100 years [10].

Older age is the biggest risk factor for AD [11] yet many centenarians, individuals who have surpassed the average life expectancy of their peers by more than two decades, avoid pathologically defined AD and/or clinical dementia [12,13]. Meta-analyses of dementia prevalence among older adults have found rates of about 1 to 3% among individuals aged 65 to 69 years with an exponential rise with increasing age bands [14] that levels off at about 40% after age 95 [15]. Most centenarian studies report slightly higher dementia prevalence rates around 60–70%, however, there is wide variation most likely dependent upon methodology and population ascertainment (see [16] for review). Yet, more consistently across studies, results indicate that not all centenarians have dementia and, perhaps even more significant, about one quarter of the population has no cognitive impairment [17-22].

The exceptionality of centenarians (i.e., their extreme survival), is the reason that they are a powerful cohort among which to examine genetic contributions to longevity and healthy aging. The sensitivity of a genetic risk model to correctly classify individuals as long-lived increased with increasing age exceptionality (i.e., 71% specificity in classifying individuals aged >102 years and 85% specificity in classifying individuals aged >105 years) indicating that the genetic contribution to longevity becomes stronger when looking at older ages [23]. The ability to reach exceptional ages without an age-related disease is also considered an extreme phenotype which can increase the power to identify genetic variants associated with a reduced risk of disease. Using centenarians as extreme controls against cases with specific age-related diseases has been shown to increase the power to detect associations between genetic variants and risk of disease for type II diabetes [24] and AD [25].

Unexpectedly it seems that centenarians do not achieve their exceptional longevity due to the absence of genetic variants associated with disease, as centenarians have been found to have variants related to increased risk of cancer, cardiac disease, and even neurodegenerative diseases [26]. Rather, it seems that centenarians are enriched with protective genes, including variants related to a reduced risk of cardiovascular disease and hypertension [27] as well as enhanced immunity and metabolism [28]. Genetic comparisons with centenarians may also be helpful in evaluating the clinical significance of genetic variants found to be associated with disease as those that are present in centenarians clearly do not preclude long survival [26].

## CENTENARIANS AS MODELS OF RESISTANCE TO AD

Individuals with resistance to AD exhibit lower than expected levels or even the complete absence of AD pathology [1], namely beta-amyloid deposition and pathologic tau [29]. As beta-amyloid levels and tau severity have been shown to increase with age [30], it is expected that those at advanced ages, such as centenarians, would have significant levels of beta-amyloid and tau. More specifically, neuropathological studies have found that prevalence of beta-amyloid is low among 40 year olds at 11% and rises steeply with increasing age to 74% of 80 year olds [30]. Yet among centenarians, individuals who have lived an additional two decades, 20% do not have amyloid plaques [13,30]. Centenarians with little or no beta-amyloid must therefore have mechanisms of resisting this abnormal protein deposition. Resistance to tau, however, appears less promising as neuropathological studies show that all centenarian brains have at least some neurofibrillary tangles [13,31]. However, a neuropathological study across the age range of 1 to 100 years found that it is exceptionally rare for individuals over the age of 10 years to have a complete absence of abnormal tau whereas development of beta-amyloid pathology appears to begin around 40 years and increases with age [30]. Additionally, there is debate about whether neurofibrillary tangles in the absence or scarcity of amyloid plaques may represent a different disease process than AD called primary age-related tauopathy (PART) [32,33].

In younger cohorts, identification of individuals without AD is biased because it is not known whether they may get AD in the future. A centenarian who is cognitively healthy at age 100 has a high probability of remaining cognitively healthy until death, with the exception of a terminal decline in the last few months of life [18]. However, it has been argued that very old individuals who have levels of pathology not meeting criteria for AD merely reflect a preclinical stage of the AD neurodegenerative process [34]. Although this may be the case for some centenarians, even the ability to delay preclinical stages of AD to 100 years and beyond demonstrates resistance to AD. Since it has been estimated that interventions leading to 5 year delay in the onset of AD would reduce the prevalence by 50% [35], understanding the mechanisms contributing to centenarians’ ability to delay onset could have a significant impact for the general population. Cognitively healthy centenarians who upon neuropathological examination do not have evidence of AD pathology, are ideal candidates for identifying genetic and environmental factors conferring resistance to AD.

The genetic variant that is most significantly associated with AD is the *APOE* gene. Having one copy of the e4 allele is associated with a 3.7 greater odds of developing AD compared to individuals who are homozygous for e3 [36] (see [37] for review). In contrast the e2 allele confers some protection against AD as individuals with one copy of the e2 allele have an odds ratio of 0.6 for development of AD in comparison to those who are e3/e3 [36]. Among centenarians there is an increased prevalence of the e2 protective allele, and more specifically the e2/e3 genotype, and a decreased prevalence of the e4 risk allele in comparison to controls [38-40]. This enrichment of a protective genetic factor among centenarians has already been harnessed to learn about serum protein profiles associated with the *APOE* e2 allele which identified proteins involved in inflammation, accumulation of beta-amyloid, and cell death [41]. This protein signature was able to differentiate individuals with AD from healthy controls as well as individuals with different longitudinal cognitive trajectories suggesting that these proteins may be valuable molecular targets for protection against AD for the general population. Additional studies to identify the protective mechanism underlying the *APOE* e2 allele are needed.

Alternatively, centenarian cohorts may also serve as control groups for the study of AD. Genetic variants with roles in lipid and cholesterol metabolism (e.g., *APOE*) and immunological processes that were identified in large GWAS studies were replicated in a case-control study comparing individuals with AD to cognitively healthy centenarians [25]. Similarly, centenarians were shown to have higher expression of sirtuin 1, a brain enzyme believed to play a role in synaptic plasticity and neuroprotection from AD, when compared to both younger controls and individuals with AD [42]. In addition to replicating findings of genetic and molecular correlates of neuroprotection from AD, these studies show that the exceptionality of centenarians as healthy agers increases the power to detect effects even with smaller sample sizes. For example, the effect sizes in Tesi et al. [25] were increased up to 6-fold compared to studies not using centenarians as controls. Moreover, it is possible that some subsets of centenarians may serve as even more robust models of resistance to AD such as centenarians who have additional risk factors for AD and amyloid (e.g., an *APOE* e4 allele) or cognitively healthy supercentenarians, who significantly delay the onset of cognitive impairment even beyond younger centenarians [2].

Studies of centenarians who are resistant to AD may reveal mechanisms underlying the ability to ward off neurodegenerative processes and cognitive dysfunction even at extreme old ages. Lifestyle habits of centenarians may be associated with better clearance of beta-amyloid as longer sleep duration is associated with less beta-amyloid accumulation [43] and more than half of centenarians in one study reported sleeping 8 hours or more per night at age 70 years [44]. Other findings might include genetic variants and other behavioral contributors, such as participation in cognitively stimulating leisure activities that preserve brain structure and function (i.e., brain maintenance, see [45] for review) even in the context of extreme aging.

## CENTENARIANS AS MODELS OF RESILIENCE TO AD

The concept of resilience has been broadly defined as the ability to effectively adapt to significant sources of stress and avoid an adverse outcome [46]. In reference to AD, individuals who demonstrate resilience are better able to cope with AD pathology, or in other words, they have a higher level of pathology (i.e., beta-amyloid and tau) in their brains than would be expected based on their cognitive function or brain structure [1]. In other cohorts it may be difficult to distinguish between resistance and resilience, or whether individuals have a limited progression of AD pathology versus better coping with increased pathology. As centenarians are at the end of life, neuropathological studies can confirm the presence of pathologies among individuals who are cognitively healthy to differentiate those who avoid pathology versus those who cope better with it. For example, in a study of 40 centenarians who self-reported as cognitively healthy, Ganz et al. [31] found evidence of atherosclerosis and neurofibrillary tangles among all centenarians and amyloid plaques in 92% suggesting that the centenarians in this sample were demonstrating cognitive resilience, or better than expected cognitive function considering the presence of AD and other pathology [1]. In comparison to community cohorts such as the Religious Orders Study and Rush Memory and Aging Project which found that 43% of cognitively healthy individuals had AD pathology and 72% had vascular pathology [47], centenarians may more commonly express resilience to AD and other pathology. Similar to cognitively healthy centenarians, resilience to AD has also been seen among individuals with a genetic predisposition for AD (i.e., an e4 allele of the *APOE* gene). Individuals with one e4 allele have a three times greater risk for developing AD [48] and a two to three times higher prevalence of amyloid [49] yet about 30–40% of these individuals are able to avoid clinical dementia throughout life [50,51]. These two models of resilience to AD have the potential to reveal both common and novel mechanisms underlying AD and other neurodegenerative processes as age and APOE genotype have shown both overlapping and unique associations with pathological markers [52]. As such, a particularly robust model of resilience to AD may be centenarians who have an e4 allele thus possessing two risk factors for AD.

Evidence for cognitive resilience specifically to AD among centenarians stems from the disassociation of AD pathology and clinical cognitive outcomes at very old ages. There is a decreased association of amyloid plaques and neurofibrillary tangles with the presence of dementia with increasing age, whereas vascular pathology (e.g., infarcts, lacunes, and small vessel disease) and cortical atrophy remain strong predictors [53]. More specifically, among individuals aged 95+ years, those without dementia, who would be expected to have low levels of AD pathology, had similar levels of AD pathology as those with dementia. Similarly, Gold et al. [54] found that levels of neurofibrillary tangles that are sufficient for dementia in adults are not sufficient among individuals over the age of 90 years, further suggesting a clinic0pathologic disconnect at very old ages. It may be that other pathologies play a greater role in cognitive impairment among individuals with exceptional longevity. Cognitive performance of self-reported cognitively healthy centenarians had a greater positive association with neurofibrillary tangle burden and granulovacuolar degeneration than with beta-amyloid pathology [31]. Additionally, as the effect size of the associations were small, the coexistence of multiple pathologies may play a greater role in determining cognitive function among centenarians than single pathologies.

Whereas most evidence for centenarians’ resilience to AD pathology has centered around cognitive resilience, some centenarians may also demonstrate brain resilience, or structural integrity of the brain that is better than expected for the level of AD pathology [1]. For example, although Ganz et al. [31] found evidence of pathologies that are associated with cognitive impairment in 100% of the centenarian brains within their sample, 50% had no signs of atrophy. This is striking because age-associated trends in gray matter volume have revealed a 5% decrease per decade after age 70 years among cognitively healthy individuals [55]. Therefore, this subset of centenarians may have been able to ward off the harmful downstream effects of neuropathologies and age-related changes including neuronal loss or change in brain structure.

Perhaps one impediment to studying brain resilience among centenarians is the need for longitudinal assessments to understand the temporal relationship between deposition of AD pathology and structural or functional brain changes. Whereas cognitive performance is generally regarded to be a downstream behavioral manifestation of brain substrate and pathological processes [56], it is not as clear whether all changes to brain structure and function occur downstream of AD pathology. Rather, they could potentially be reflecting an alternate pathological process that is distinct from AD [57]. Cognitive function and therefore cognitive resilience are also easier to measure than brain resilience among centenarians as they require only paper and pencil testing whereas in-vivo measures of brain structure and network integrity are not as feasible to collect in centenarians who often have mobility impairments that complicate travel to neuroimaging centers, and are less able to tolerate long scanning protocols.

Mechanisms underlying resilience to AD among long-lived individuals may include factors that confer brain reserve and/or cognitive reserve [58]. Brain reserve refers to individual differences in brain structure or brain processes (e.g., larger intracranial volume, greater synaptic density, or higher rates of neurogenesis) [58,59] which allow for greater tolerance to pathology before resultant functional consequences (e.g., cognitive impairment) emerge [60]. In contrast, cognitive reserve pertains to individual differences in how the brain copes with pathology including more efficient use of existing cognitive networks (i.e., neural reserve) or the ability to recruit alternate networks in response to network disruptions (i.e., neural compensation) [61,62]. Comparisons of centenarians who are resilient to AD pathology with referent cohorts, such as individuals who have dementia even with low levels of AD pathology have the potential to identify protective and risk factors such as genetic variants related to differences in brain structure or function, lifestyle habits that increase or decrease thresholds for cognitive impairment caused by AD pathology, and behaviors that strengthen or weaken alternate neural networks.

## CENTENARIANS AS MODELS FOR DISCOVERING OTHER CONTRIBUTORS TO AD AND RELATED DEMENTIAS

Among all age groups there is significant heterogeneity in what clinically may appear to be AD due to variations in impaired cognitive functions, patterns of atrophy and functional activation, progression of the disease, and other coexisting pathologies (see [63] for review). The greater availability of neuropathological data among centenarians due to their high mortality rate is a potential opportunity for studies of other contributors to dementia because it allows for the evaluation of pathologies beyond what can be obtained from cerebrospinal fluid and imaging (e.g., beta-amyloid, tau, and vascular disease). Furthermore, although AD pathology is not uncommon among the oldest individuals, it appears that rarer pathologies become more common with increasing age. For example, after age 95 years prevalence of hippocampal sclerosis pathology increases whereas the prevalence definite AD pathology decreases [64]. In a neuropathological examination of 77 centenarian brains there was evidence of a variety of neuropathologies: 21% had hippocampal sclerosis, 17% had Lewy body disease, 27% had TDP-43 pathology, and small vessel disease and infarcts were common [13]. Other studies have also found high rates of cerebral amyloid angiopathy (77%) [31] argyrophilic grain disease (31%) [65] and alpha-synucleinopathy (35%) [66] whereas some neurodegenerative diseases, such as frontotemporal lobar degeneration are not prevalent at advanced ages [13]. However, a review of community-based studies of neuropathology showed wide variation in prevalence rates of these pathologies among older adults with rates of 3–13% for hippocampal sclerosis, 13–46% for TDP-43, 6–39% for Lewy body pathology, and 28–70% for vascular pathologies [67] indicating that additional studies are needed to determine the pathologies that are most associated with cognitive impairment at extreme old ages.

Although rare, neuropathological studies have identified a subset of centenarians without evidence of any pathological process: Neltner et al. [13] found 3 cases (4%) without evidence of significant neurofibrillary tangles, cerebral amyloid angiopathy, arteriolosclerosis, hippocampal sclerosis, TDP-43 pathology, Lewy body disease, or large infarcts whereas Ding et al. [65] identified 6 centenarians (19%) without AD, Parkinson’s disease, infarcts, white matter lesions, vascular dementia, dementia with tangles, or argyrophilic grain disease. This subset of centenarians may represent individuals who are resistant to all forms of neuropathologies underlying cognitive impairment and dementia. If indeed this is true, these centenarians may serve as controls for a variety of neurodegenerative diseases and unhealthy agers to identify novel risk or protective factors. However, it is also possible that other pathologies may have been present in the brains of these centenarians that either were not tested for or that have not yet been discovered, paving the way for discoveries of new biomarkers of existing diseases or pathological mechanisms of new diseases.

## CENTENARIAN OFFSPRING FOR STUDYING LIFE COURSE CONTRIBUTORS TO RESISTANCE OR RESILIENCE

Longevity runs in families such that, in comparison to controls, siblings of centenarians have a nine times greater chance of becoming centenarians themselves [68] and the offspring of centenarians have a 62% lower risk of mortality compared to age-matched controls [69]. This implies a strong familial component consisting of genetic and behavioral or environmental contributions to the ability to reach extreme old ages. Furthermore, whereas heritability of average lifespan is about 10–25% [70,71], heritability of reaching ages well beyond average life expectancy (i.e., 100 years) appears to be about 30–50% [72].

Studies of familial longevity, such as the NIA-funded Long Life Family Study, have found that in addition to increased survival, family members of long-lived individuals appear to have longer health spans, as demonstrated by longer disease-free survival, than referent populations [73,74]. The predisposition for healthy aging among family members of long-lived individuals has led centenarian studies such as the New England Centenarian Study, the Longevity Genes Project, the Chinese Longitudinal Healthy Longevity Survey, the Tokyo Centenarians Study, and the Japanese Semi-supercentenarians Study to expand to include the offspring of their centenarian participants. These and other studies have found that centenarian offspring have a reduced risk and delayed onset of age-related diseases including heart disease, hypertension, diabetes, and stroke in comparison to offspring of parents who did not reach exceptional ages [75]. They also have more favorable health characteristics demonstrating better lipid profiles [76], lower markers of inflammation [77], lower body mass index, and better physical function [78], mental health, and well-being [79].

Of relevance to studies of AD and related dementias, our work at the New England Centenarian Study has found that centenarian offspring have a 46% lower prevalence of cognitive impairment compared to controls at a mean age of 75 years and a 27% reduced risk of becoming impaired over follow up [80]. This suggests that centenarian offspring have an extended cognitive health span in comparison to their peers and therefore may be a valuable cohort for assessing the contribution of healthy life spans to maintaining good cognitive function and the avoidance of AD pathology without the confounds that exist among some centenarians including sensorimotor deficits and chronic late-life illnesses. As centenarian offspring are earlier in their life course than centenarians, they also afford the opportunity to evaluate the role of midlife vascular risk factors such as hypertension, type II diabetes, obesity, dyslipidemia, smoking, and physical inactivity on risk of AD. In line with this notion, Arai et al. [77] found that markers of inflammation predicted cognition among older adults yet centenarians, on average, have higher levels of these inflammation markers than younger individuals. In contrast, their offspring had lower levels of inflammation in comparison to spouses suggesting a life-long lower level of inflammation that may be obscured by end of life changes among centenarians.

Assessment of behavioral and lifestyle factors that may delay the onset or change the risk of AD throughout older adulthood such as engagement in cognitively stimulating leisure activities, occupational complexity, diet, physical activity, and psychosocial characteristics may be better achieved through studying centenarian offspring. These factors can be difficult to assess among centenarians due to late life changes in behavior secondary to frailty and sensorimotor impairments and inaccurate recall for retrospective data collection. Some studies such as the New England Centenarian Study and the Longevity Genes Project have collected data on behavior and lifestyle of centenarian offspring [44,81-83] that could be integrated with cognitive data and dementia outcomes to better understand how these factors change the risk of cognitive impairment and AD among healthy agers. These and other studies of centenarian offspring should further implement longitudinal data collection of behavioral and psychosocial factors along with cognitive assessments to be able to identify causal pathways.

In spite of evidence that centenarian offspring demonstrate healthy cognitive aging, to date there are no studies of biomarkers of AD pathology in this cohort. Post-mortem neuropathological studies are less feasible among centenarian offspring than their centenarian parents because they are often still many years from death, therefore, biomarkers of AD pathology should be obtained by assays of cerebrospinal fluid at a minimum or ideally, in vivo imaging including magnetic resonance imaging (MRI), diffusion tensor imaging (DTI), and positron emission tomography (PET) for amyloid and tau proteins. These studies would be valuable for identifying offspring who are on resistant (i.e., absence of AD pathology without clinical cognitive impairment) or resilient (i.e., presence of AD pathology without cognitive impairment) trajectories with longitudinal studies providing additional power to identify mid-life contributors to healthy cognitive aging.

Additionally, finding an appropriate referent group for centenarians has long been a challenge. Comparing centenarians to younger individuals introduces the possibility that differences in health and function are actually due to differences in life experiences or exposures that are specific to each birth cohort. Their peers, members of the same birth cohort who did not live long lives, passed away decades ago. Some long-term prospective cohort studies such as the Health and Retirement Study or the Framingham Heart Study have peer referents for centenarians, however the sample sizes of centenarians in their cohorts are small owing to the fact that centenarians are rare, comprising only 0.08% of individuals age 65 years or older worldwide [84]. Studying the offspring of centenarians provides the advantage that their peers are still alive. Commonly used referent groups for centenarian offspring include the spouses of the offspring as well as offspring of individuals who died at average life expectancy for the birth cohort (e.g., offspring of individuals who died in their 70s). Perhaps an unexpected referent group are the siblings of centenarian offspring. As these offspring usually had only one long-lived parent, not all of the offspring will have longer than average health spans because of the contributions of their non-centenarian parent. Therefore, enrolling multiple offspring of the same family allows for the comparison of discordant siblings, e.g., those who do not show cognitive decline or pathological brain changes versus those who do, to identify protective and risk factors for cognitive aging and AD. However, the tradeoff in studying offspring is that they are also subject to cohort effects as they were born in a different cohort than the centenarians and it is likely that some factors that enabled centenarians to achieve resistance or resilience to AD may not be seen among their offspring.

### Theoretical Models of Resistance and Resilience among Centenarian Offspring

**Figure 1.**
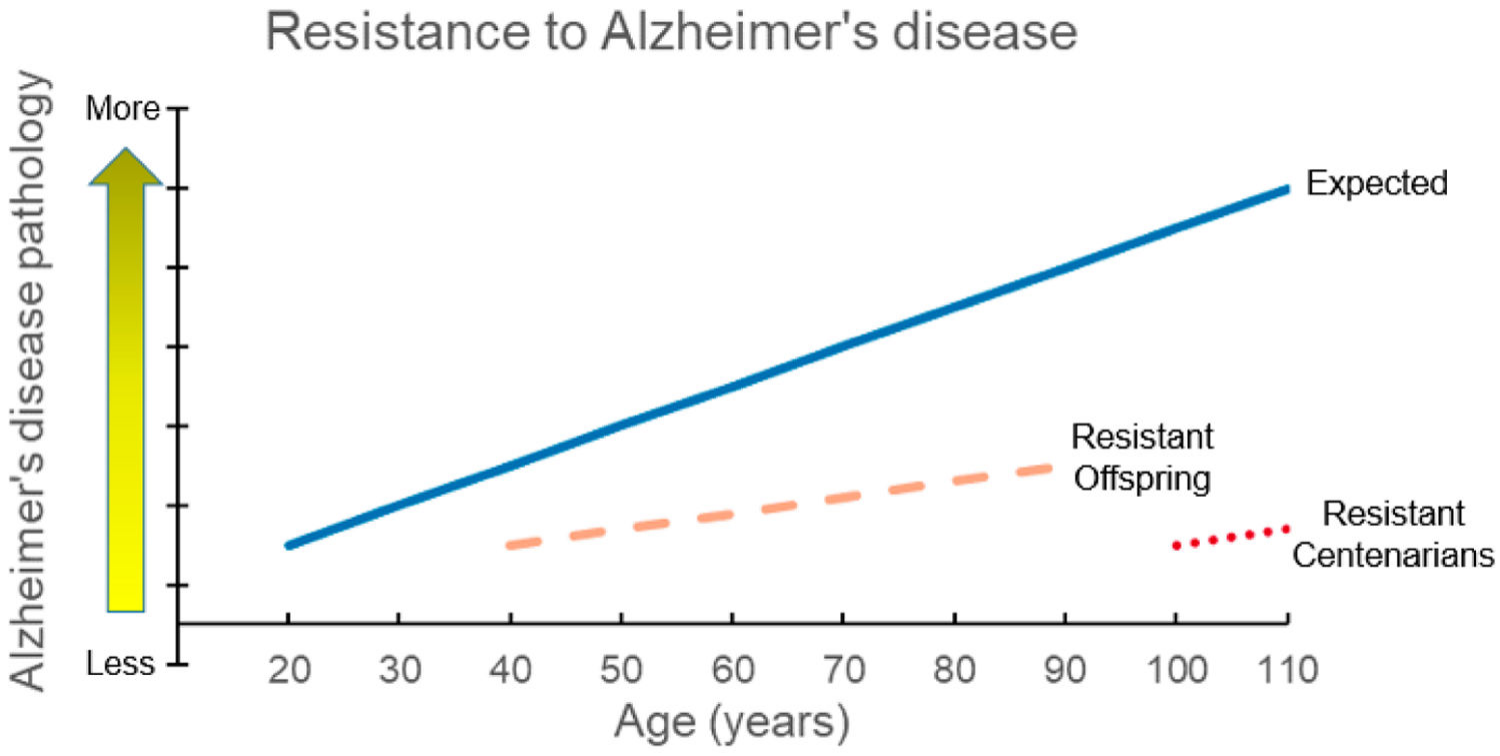
Centenarians and centenarian offspring who are resistant to Alzheimer’s disease (AD). This theoretical model shows the expected increase of AD pathology with increasing age (blue solid line). Centenarians who are resistant to AD and therefore avoid both clinical and pathological hallmarks of AD are expected to have negligible levels of AD pathology at the extreme ages of 100+ years (red dotted line). Centenarian offspring who are resistant to AD (orange dashed line) are expected to have a muted effect of resistance to AD due to receiving only half of their genetics from their centenarian parent. This model also demonstrates the added advantage of being able to study centenarian offspring across a wider range of older adulthood relative to centenarian cohorts using longitudinal assessments.

Studies of centenarian offspring have the potential to characterize long-term trajectories of cognitive function and AD pathologic change. Because centenarians are at the end of their life spans, longitudinal follow up is highly limited and therefore analyses are restricted to cross-sectional associations between predictors and outcomes rather than causal inferences. Longitudinal studies of centenarian offspring would allow us to better describe progression of neuropathological changes including amyloid deposition, vascular insults, and structural changes in relation to changes in cognitive function. This would facilitate differentiating between individuals who have a slowed progression of neuropathological changes and are therefore demonstrating resistance to AD versus those who are maintaining good cognitive function in spite of existing pathology and are therefore demonstrating resilience. As shown in Figure 1, the subset of centenarian offspring who are resistant to AD pathology would be those who maintain low levels of beta-amyloid and tau burden in spite of increasing age or in comparison to age-matched referents as ascertained during assessments from midlife through late adulthood. These longitudinal assessments would provide meaningful data for identifying not only individuals who stay on the trajectory of resistance but also for capturing the time point of divergence for those who are not resistant or who lose resistance and the related temporal factors such as changes in health, cognition, or lifestyle.

**Figure 2.**
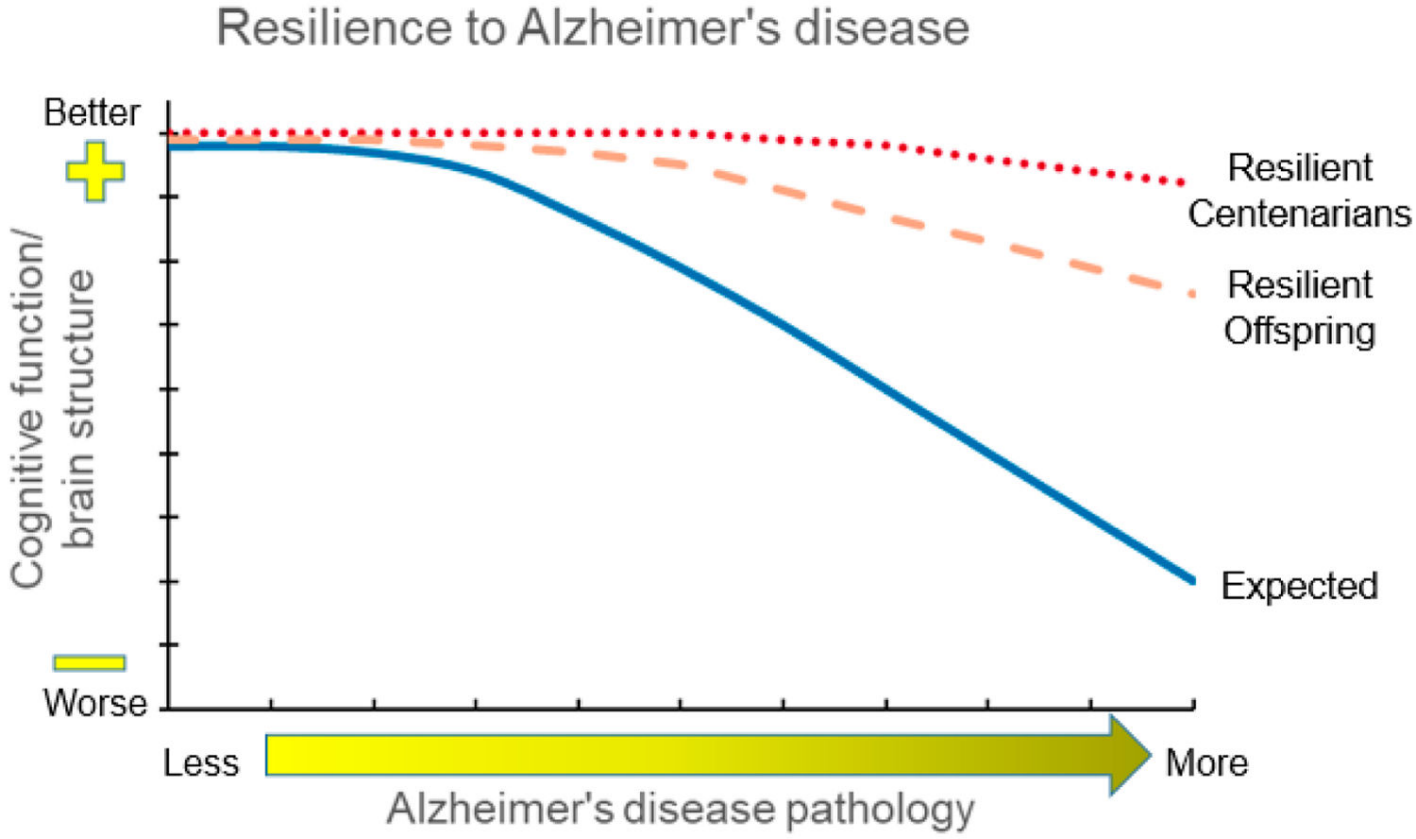
Centenarians and centenarian offspring who are resilient to Alzheimer’s disease (AD). This theoretical model shows the expected declines in cognitive function and/or brain structure integrity associated with increasing levels of AD pathology (blue solid line). Centenarians who are resilient to AD pathology (red dotted line) are expected to exhibit no or only minimal functional and structural changes despite high levels of AD pathology, whereas centenarian offspring who are resilient to AD pathology (orange dashed line) are expected to have a muted effect of resilience due to receiving only half of their genetics from their centenarian parent.

In contrast, a separate subset of centenarian offspring who demonstrate resilience to AD, or better coping to AD pathology, would be those who maintain good cognitive function and structural integrity of the brain in spite of increasing AD pathologic burden as shown in Figure 2. Comparisons of offspring who demonstrate resilience with referents who show expected correlations between cognitive function and AD pathologic burden may help to identify mechanisms underlying resilience, such as brain reserve and cognitive reserve as described above for centenarian resilience. Additionally, we may also be able to identify the shape of the trajectory of AD pathologic burden to better understand whether long-lived individuals with AD pathology at death had a very slow progression of AD pathology throughout a long life or a long delay in the onset of AD pathology with beta-amyloid deposition occurring rapidly in the last years of life.

## THE FUTURE OF CENTENARIAN STUDIES

There has been recent growth in the field of geroscience, which aims to understand how and why basic aging mechanisms contribute to most, if not all, chronic age-related diseases in order to prevent, cure, or delay chronic age-related diseases in tandem rather than following the prevailing paradigm of curing one disease at a time (see [85] for review). This has not surprisingly spurred interest in centenarians who delay or escape age-related disease and are therefore models of healthy aging. Studies funded by the National Institute on Aging including the Longevity Consortium [86] and the Integrative Longevity Omics Project (http://www.longevityomics.com) are currently focused on collecting “deep phenotyping” data, consisting of detailed physical and cognitive function assessments, behavioral and psychosocial data, and biological specimens (i.e., blood and fecal samples) from centenarians. These efforts will pave the way for future studies of genetic and multi-omics data including proteomics, transcriptomics, methylomics, metabolomics, and microbiomics to identify genetic and molecular signatures associated with exceptional longevity and healthy aging and to integrate these findings with cognitive and behavioral data. The data from these studies and particularly from subsets of centenarians who are cognitively healthy will be immensely valuable in extending our understanding of the mechanisms and pathways underlying resilience and resistance to AD.

## CONCLUSIONS

In spite of having one of the greatest risk factors for AD, advanced age, some centenarians are able to avoid cognitive dysfunction caused by AD pathology or entirely avoid the pathology itself. Some of this may be due to the longer health span of centenarians which includes avoidance of other risk factors for AD such as midlife vascular risk factors and late-life chronic conditions as well as the potential contributions of cognitive and brain reserve. Studying centenarians and their family members who are also predisposed to longer cognitive health spans has the potential to reveal pathways and mechanisms that confer protection from AD pathology and cognitive impairment due to AD pathology. The hope is that these studies will identify genetic, environmental, behavioral, and life course contributors to AD prevention that will be developed into interventions for healthy cognitive aging for the general population.
